# Closing the health gap in Central Australia: reduction in Indigenous Australian inpatient self-discharge rates following routine collaboration with Aboriginal Health Workers

**DOI:** 10.1186/s12913-023-09921-7

**Published:** 2023-08-17

**Authors:** Tim Cheok, Morgan Berman, Richard Delaney-Bindahneem, Matthew Phillip Jennings, Linda Bray, Ruurd Jaarsma, Pradeep Mathew Poonnoose, Kanishka Williams, Narlaka Jayasekera

**Affiliations:** 1 Department of Trauma and Orthopaedics, Alice Springs Hospital, 6, Gap Road, The Gap, Northern Territory, 0870 Australia; 2 Department of Orthopaedic Surgery, Palmerston North Hospital, 50, Ruahine Street, Roslyn, Palmerston North, 4414 New Zealand; 3 Department of Orthopaedic Surgery, Monash Medical Centre, 246, Clayton Road, Clayton, Victoria 3168 Australia; 4 Department of Plastics and Reconstructive Surgery, Bendigo Base Hospital, 100 Barnard Street, Bendigo, VIC 3350 Australia; 5 Aboriginal Liaison Services, Alice Springs Hospital, 6, Gap Road, The Gap, Northern Territory, 0870 Australia; 6 Department of Orthopaedic Surgery, Flinders Medical Centre, Flinders Drive, Bedford Park, South Australia 5042 Australia; 7 Department of Orthopaedic Surgery, Christian Medical College Hospital, IDA Scudder Road, Vellore, Tamil Nadu 632004 India; 8 Department of Orthopaedic Surgery, Wairau Hospital, Hospital Road, Blenheim, 7201 New Zealand

**Keywords:** Indigenous health, Self-discharge, Surgery, Orthopaedics

## Abstract

**Background:**

Indigenous Australians experience significant socioeconomic disadvantage and healthcare disparity compared to non-Indigenous Australians. A retrospective cohort study to describe the association between rates of self-discharge in Indigenous orthopaedic patients and the introduction of routine Aboriginal Liaison Officers (ALO) within the Orthopaedic multi-disciplinary team (MDT) was performed.

**Methods:**

ALO were introduced within our routine Orthopaedic MDT on the 22^nd^ of February 2021. Two patient cohorts were analysed, Group 1; patients admitted in the 9-months prior to inclusion of ALO, and Group 2; patients admitted within 9-months thereafter. The primary outcome of interest was the rate of self-discharge among Indigenous patients. Secondary outcomes of interest were the stage of treatment when patients self-discharged, recurrent self-discharge, risk factors for self-discharge and association between self-discharge and length of hospital stay.

**Results:**

Introduction of ALO within routine Orthopaedic MDT was associated with a significant 37% reduced risk of self-discharge among Indigenous patients (*p* = 0·009), and significantly fewer self-discharges before their definitive surgical and medical treatment (*p* = 0·0024), or before completion of postoperative intravenous antibiotic treatment (*p* = 0·030). There was no significant change in the risk of recurrent self-discharge (*p* = 0·557). Risk factors for self-discharge were younger age; pensioners or unemployed; residents of Alice Springs Town-Camps or of communities within 51 to 100 km of Alice Springs; and those diagnosed with lacerations of the upper limb, but without tendon injury, wound and soft tissue infections or osteomyelitis. In Group 2, the odds of self-discharge decreased with increased length of hospital stay (*p* = 0·040).

**Conclusions:**

Routine inclusion of ALO within the Orthopaedic MDT reduced the risk of self-discharge in Indigenous patients. Those who self-discharged did so only after critical aspects of their care were met.

## Introduction

Indigenous Australians experience significant socioeconomic disadvantage and healthcare disparity compared to non-Indigenous Australians [1]. Historically, generations of Indigenous Australians have faced rampant systemic discrimination since British colonialisation. Between 1883 and 1969, Indigenous children were forcefully removed from their families by the Australian Government and church missionaries, with victims of this period coined the “Stolen Generation” [2, 3]. Cumulative generational trauma among Indigenous Australians has resulted in their social disenfranchisement, poor health literacy, and impaired self-advocacy [1].

Self-discharge describes events where patients leave a health service before discharge by their clinician [4]. Data from the Australian Institute of Health and Welfare showed that the age-standardised rate of self-discharge among Indigenous patients had risen from 11 to 16 per 1,000 population between 2004–05 to 2016–17 [5]. These figures are of significant concern as patients who self-discharge from hospital are more likely to be readmitted and succumb to higher rates of morbidity and mortality [6]. Self-discharge is associated with patient dissatisfaction and negative hospital experience, and hence considered a surrogate measure of patient satisfaction [7]. There is no consensus definition of self-discharge in the literature [8]. For this study, we defined self-discharge as patients who had left the service for 24 h without prior agreement with the treating team.

Alice Springs Hospital (ASH) is the major referral centre for Central Australia, with a catchment area of over 1.2 million square-kilometres, and a population of approximately 42,000 [9]. Roughly 60% of the population reside within the town of Alice Springs, and the remaining population are widely dispersed between the rural town of Tenant Creek and fifty other rural communities, some of which extends into the adjacent states of South Australia and Western Australia. As many as seventeen different languages are spoken by Central Australia’s Indigenous people [10, 11], and is often as their first and preferred language. To improve communication with this diverse population, the government inducted Aboriginal Health Workers to help with health care delivery. Though they have been an integral part of the Northern Territory workforce since the 1870s, this rich, varied, and accessible resource have not been engaged to their full capacity [12, 13].

Research by Einsiedel et. al. in Alice Springs has emphasised the dire need for better communication among clinicians, patients, and other healthcare providers. They found 73·4% of self-discharged patients from medical units had done so with little understanding of their diagnosis or their expected trajectory of disease [14]. Routine use of Aboriginal Health Workers in Australia is poorly described in literature [8]. A qualitative study by Kerrigan and colleagues showed improved communication and patient satisfaction with consistent utility of interpreters [15]. To improve communication, and provide effective health care, the Orthopaedic team at ASH incorporated Aboriginal Liaison Officers (ALO), part of the broader family of Aboriginal Health Workers, into the routine care of Indigenous patients. In this study, we aim to demonstrate the effect of regular ALO presence within the Orthopaedic multi-disciplinary team (MDT) on the rates of self-discharge among Indigenous orthopaedic patients. We also explore the stage of treatment at which patients self-discharge, risk factors for self-discharge, and the association between length of stay and self-discharge.

## Materials and methods

This retrospective cohort study was conducted at a single centre. Ethical approval was obtained from the Central Australian Human Research Ethics Committee prior to commencement of this study (Approval No.: CA-22–4376). Reporting was performed based on the recommendations by Strengthening the Reporting of Observational Studies in Epidemiology (STROBE) [16].

### Study design

A retrospective analysis of case-notes of patients admitted to the Orthopaedic unit at ASH was performed. Patient episodes were divided into two groups. Group 1 were patients admitted during the 9-month period between 23^rd^ of May 2020 and 22^nd^ of February 2021. These patients were seen by ALO only if their service had been requested by the treating team. This *ad-hoc* utility was suspected suboptimum by clinicians within our unit. Group 2 were patients admitted between 23^rd^ of February and 22^nd^ of November 2021. Regular ALO services were incorporated into the Orthopaedic MDT from the 22^nd^ of February 2021, and since then, they have been integral to the daily morning meetings, where patient admissions and proposed management are discussed. ALO also attended subsequent daily morning ward-rounds and acute emergency department patient assessments during business hours of weekdays. Additional ALO engagement was sought throughout the day as required.

### Setting and intervention

This study was performed in ASH, a publicly funded hospital within the Northern Territory of Australia. It is the primary referral centre for Central Australia, serving a population of approximately 42,000 people, approximately one-third of which are Indigenous Australians [9]. The Orthopaedic service at ASH provides treatment for patients presenting to Tennant Creek Hospital, a separate regional hospital, 508 kms away from Alice Springs, and to over 50 other rural communities in Central Australia. Patients from Tennant Creek Hospital are currently required to attend ASH for acute and elective treatment, and outpatient follow-up when telehealth is deemed unsuitable. Within the current framework of our service, ALO are available only during business hours on weekdays. There is no ALO service provision on weekends or public holidays.

### Patient characteristics and outcomes measured

Baseline characteristics, including age, gender, marital status, employment status and place of residence, as well as diagnosis and length of stay were recorded for each hospital episode. Patients were classified as Indigenous or non-Indigenous based upon self-identification at time of admission. Residence was categorised as either (1) Alice Springs and suburbs, (2) Town-Camps within Alice Springs, (3) Tennant Creek, (4) communities less than 50 kms of Alice Springs, (5) communities 51 to 100 kms of Alice Springs, (6) communities 101 to 300 kms of Alice Springs, (7) communities 301 to 500 kms of Alice Springs, (8) communities further than 500 kms of Alice Springs and (9) residence beyond the catchment area of Alice Springs Hospital. Alice Springs Town-Camps are Indigenous communities within the town of Alice Springs, where each Town-Camp title is held by a Housing Association under a perpetual lease [17].

The primary outcome of interest was the rate of self-discharge amongst Indigenous patients. Patients were prospectively classified as a ‘self-discharge’ if they left the ward for more than 24 h without the prior agreement of the managing clinical team. This cut-off was established following consultation with stakeholders within our healthcare service (medical administration, clinicians, and Indigenous health representatives). For this study, patients who left against medical advice, were not considered “self-discharge”. In this scenario, the treating team has explained the risks of discontinuing their recommended treatment, and where appropriate, provided an alternative treatment plan acceptable to the patient. Therefore, unlike self-discharge, leaving against medical advice although not ideal, reflects a partnership between clinicians and patients, whilst upholding patient autonomy over healthcare decisions.

Secondary outcomes of interest were the stage of treatment attained at the time of self-discharge and the incidence of recurrent self-discharge for the same condition. For this study, we classified the stage of treatment at which patients self-discharged as either that prior to (1) definitive surgical or medical treatment, (2) completion of intravenous antibiotic course after surgery, (3) allied health clearance, or (4) collection of discharge script/ medications. If patients had more than one self-discharge episode for the same problem, we classified the treatment stage based on the first episode of self-discharge. Multiple self-discharges for the same condition was treated as a single episode for our analysis. We also performed analysis to determine risk factors for self-discharge, using baseline and admission characteristics. Lastly, we measured the association between length of stay and self-discharge.

All Indigenous patients admitted during the study period were included in this analysis. We also performed a brief analysis of non-Indigenous patients admitted within the same time-period, to provide context and comparison between Indigenous and non-Indigenous patients.

### Statistical analysis

Categorical variables were displayed as frequency (percentage) and compared using Chi-Squared test. Continuous variables were displayed as mean ± standard deviation and compared using unpaired t-test. Logistic regression utilising a penalised maximum likelihood model, as described by Firth [18], was performed to delineate risk factors for self-discharge. This was chosen due to separation encountered after attempting a conventional binomial model [19]. To determine the association between self-discharge and length of stay, a conventional logistic regression analysis was performed, as we did not encounter any separation. All statistical analyses were performed using Stata Version 15.1 (StataCorp, USA). The threshold for statistical significance in this study was set at 95%.

## Results

Between 23^rd^ of May 2020 and 22^nd^ of November 2021, a total of 2,147 patient episodes admitted under our Orthopaedic service was identified. 1,135 Indigenous patient episodes were included in our primary analysis. The remaining 1,012 non-Indigenous patient episodes were analysed for comparison.

There were 617 patients in Group 1 and 518 patients in Group 2. The average age of patients was 31·06 ± 17·01 and 31·10 ± 18·19 years in Groups 1 and 2 respectively. This was not statistically significant. In both groups, most patients were male, never married, and unemployed. There was no significant difference between groups in terms of gender, marital status, employment status or place of residence. Majority of patients presented with an acute presentation, most commonly fracture or dislocation. Significantly more patients presented with tendon injuries in Group 1, though other diagnoses did not differ significantly between groups. The average length of stay was comparable in both groups. A detailed description of the baseline characteristics is shown in Table 1.

**Table 1:** Baseline characteristics of patients

	**Group 1 (*n* = 617)**	**Group 2 (*n* = 518)**	***p*-Value**
**Age (Years)**	31·06 ± 17·01	31·10 ± 18·19	0·97
**Gender**			
Male	336/ 617 (54·46%)	296/ 518 (57·14%)	0·36
Female	281/ 617 (45·54%)	222/ 518 (42·86%)	
**Marital Status**			
Married/ De-Facto	199/ 617 (32·25%)	168/ 518 (32·43%)	0·70
Separated	29/ 617 (4·70%)	19/ 518 (3·67%)	
Widowed	16/ 617 (2·59%)	18/ 518 (3·47%)	
Never Married	373/ 617 (60·45%)	313/ 518 (60·42%)	
**Employment Status**			
Employed	92/ 617 (14·91%)	89/ 518 (17·18%)	0·14
Home Duties	31/ 617 (5·02%)	11/ 518 (2·12%)	
Pensioner	10/ 617 (1·62%)	10/ 518 (1·93%)	
Student	89/ 617 (14·42%)	75/ 518 (14·48%)	
Child Not at School	48/ 617 (7·78%)	53/ 518 (10·23%)	
Unemployed	250/617 (40·52%)	204/ 518 (39·38%)	
Not Stated	97 / 617 (15·72%)	76/ 518 (14·67%)	
**Place of Residence**			
Alice Springs and Suburbs (Excluding Town Camps)	129/ 617 (20·91%)	127/ 518 (24·52%)	0·81
Alice Springs Town-Camps	35/ 617 (5·67%)	32/ 518 (6·18%)	
Tennant Creek	56/ 617 (9·08%)	47/ 518 (9·07%)	
Communities < 50 km from Alice Springs	34/ 617 (5·51%)	24/ 518 (4·63%)	
Communities 51 – 100 km from Alice Springs	25/ 617 (4·05%)	17/ 518 (3·28%)	
Communities 101 – 300 km from Alice Springs	130/ 617 (21.07%)	99/ 518 (19·11%)	
Communities 301 – 500 km from Alice Springs	121/ 617 (19·61%)	110/ 518 (21·24%)	
Communities > 500 km from Alice Springs	66/ 617 (10·70%)	47/ 518 (9·07%)	
Outside of Catchment Area	21/ 617 (3·40%)	15/ 518 (2·90%)	
**Diagnosis**			
**Acute Injuries**			
**Fracture/ Dislocations**			
Upper Limb Excluding Hands	85/ 617 (13·78%)	72/ 518 (13·90%)	0·44
Hands	66/ 617 (10.70%)	47/ 518 (9·07%)	
Lower Limb Excluding Foot and Ankle	35/ 617 (5·67%)	40/ 518 (7·72%)	
Foot and Ankle	39/ 617 (6·32%)	39/ 518 (7·53%)	
Spine and Pelvis	3/ 617 (0·49%)	5/ 518 (0·97%)	
** Lacerations With No Tendon Involvement**			
Upper Limb	105/ 617 (17·01%)	81/ 518 (15·64%)	0·74
Lower Limb	8/ 617 (1·30%)	10/ 518 (1·93%)	
** Tendon Injuries**			
Upper Limb	37/ 617 (6·00%)	19/ 518 (3·67%)	0·044*
Lower Limb	5/ 617 (0·81%)	2/ 518 (0·39%)	
** Infections**			
Wound/ Soft Tissue Infection	124/ 617 (20·10%)	90/ 518 (17·37%)	0·15
Surgical Site Infection	16/ 617 (2·59%)	2/ 518 (0·39%)	
Septic Arthritis	16/ 617 (2·59%)	21/ 518 (4·05%)	
Prosthetic Joint Infection	4/ 617 (0·65%)	1/ 518 (0·19%)	
Osteomyelitis	11/ 617 (1·78%)	10/ 518 (1·93%)	
** Paediatric Conditions**			
SCFE	1/ 617 (0·16%)	2/ 518 (0·39%)	0·30
Perthe’s	1/ 617 (0·16%)	2/ 518 (0·39%)	
Others	12/ 617 (1·94%)	23/ 518 (4·44%)	
** Elective**			
Major	12/ 617 (1·94%)	17/ 518 (3·28%)	0·22
Minor	37/ 617 (6·00%)	35/ 518 (6·76%)	
Length of Stay (Days)	3·79 ± 6·21	3.76 ± 6.24	0·92

^*^ Statistically significant finding; *SCFE* Slipped Capital Femoral Epiphysis

### Outcome measures

The incorporation of ALO within the routine Orthopaedic MDT was associated with statistically significant reduction in the rate of self-discharge from 12·97% to 8·11% [Risk Ratio (RR) = 0·63, *p* = 0·009]. Of those who self-discharged in Group 2, significantly fewer did so prior to definitive surgical or medical treatment (*p* = 0·024), or prior to completion of post-operative intravenous antibiotic treatment (*p* = 0·030). Patients in Group 2 who self-discharged did so later in their admission; 42·86% of self-discharges took place prior to allied health clearance compared to 10·00% in Group 1 (*p* < 0·001). There was no difference in the rates of patient self-discharge prior to collection of their planned-discharge scripts/ medications, or recurrent self-discharges between groups. Details of our findings are shown in Table 2.

**Table 2:** Outcome measures

	**Group 1 (*n* = 617)**	**Group 2 (*n* = 518)**	**Risk Ratio**	***p*-Value**
**Self-Discharge**	80/ 617 (12·97%)	42/ 518 (8·11%)	0·63	0·0085*
**Stage of Treatment**				
Self-discharge prior to definitive surgical or medical treatment	29/ 80 (36·25%)	7/ 42 (16·67%)		0·024*
Self-discharge prior to completion of intravenous antibiotic course after surgery	21/ 80 (26·25%)	4/ 42 (9·52%)		0·030*
Self-discharge prior to allied health clearance	8/ 80 (10·00%)	18/ 42 (42·86%)		< 0·0001*
Self-discharge prior to collection of discharge script/ medications	22/ 80 (27·50%)	13/ 42 (30·95%)		0·69
**Recurrence**	21/ 80 (26·25%)	9/ 42 (21·43%)	0·82	0·56

^*^Statistically significant finding

### Non-indigenous patient outcomes

When compared with non-Indigenous patients admitted over the same period as in Group 1, only 1·06% (5/471) self-discharged (versus 12·97% in Indigenous patients). Likewise, only 0·92% (5/541) non-Indigenous patients admitted over the same period as in Group 2 self-discharged (versus 8·11% in Indigenous patients). The RR of self-discharge in Indigenous patients compared to non-Indigenous patients was 12·21 (*p* < 0·001) within the same period as Group 1, and 8·77 (*p* < 0·001) within the same period as Group 2. There were no significant differences in self-discharge among non-Indigenous patients between the two periods (RR = 0·87, *p* = 0·826).

### Risk factors for self-discharge

Risk factors for self-discharge were younger age, patients who were pensioners or unemployed, patients residing in Alice Springs town-camps or communities between 51 to 100 kms of Alice Springs, and patients with lacerations of upper limb without tendon involvement, wound or soft tissue infections, or osteomyelitis. Minors, students and increasing age were protective factors against self-discharge.

Prior to the implemented change (i.e., in Group 1), risk factors for self-discharge include younger age, pensioners, patients residing in Alice Springs town-camps or communities between 51 to 100 kms of Alice Springs, and patients with wound or soft tissue infections, septic arthritis, osteomyelitis and Perthe’s disease. Female gender, minors and increasing age were protective factors for self-discharge. Following the implemented change in departmental practice (i.e., in Group 2) younger age, patients of communities between 51 to 100 kms of Alice Springs, and patients with wound or soft tissue infections were the only persistent risk factors for self-discharge. These are displayed in Table 3.

**Table 3:** Penalised maximum likelihood logistic regression model to assess for risk and protective factors

Baseline Characteristics	Both Groups			Group 1 Only			Group 2 Only		
	**Odds Ratio**	**SE**	***p*-Value**	**Odds Ratio**	**SE**	***p*-Value**	**Odds Ratio**	**SE**	***p*-Value**
**Age**	0·94	0·01	< 0·0001*	0·96	0·02	0·018*	0·94	0·23	0·021*
Female Gender	0·95	0·24	0·85	0·78	0·27	0·47	1·14	0·46	0·75
**Marital Status**									
Married/ De-Facto	Baseline			Baseline			Baseline		
Separated	0·10	0·15	0·12	0·14	0·22	0·19	0·42	0·65	0·58
Widowed	4·07	4·15	0·17	7·29	9·84	0·14	4·29	7·47	0·40
Never Married	0·78	0·23	0·40	0·81	0·31	0·57	0·90	0·43	0·82
**Employment Status**									
Employed	Baseline			Baseline			Baseline		
Home Duties	1·96	1·24	0·28	1·66	1·27	0·51	3·11	3·50	0·31
Pensioner	118·43	96·77	< 0·0001*	319·41	502·42	< 0·0001*	87·72	102·03	< 0·0001*
Student	0·27	0·14	0·013*	0·45	0·29	0·22	0·20	0·18	0·072
Child Not at School	0·05	0·04	< 0·0001*	0·02	0·04	0·018*	0·17	0·17	0·078
Unemployed	2·20	0·76	0·023*	1·95	0·84	0·12	3·10	1·87	0·060
Not Stated	0·06	0·08	0·057	0·04	0·08	0·08	0·25	0·38	0·36
**Residence Category**									
Alice Springs and Suburbs (Excluding Town Camps)	Baseline			Baseline			Baseline		
Alice Springs Town Camps	3·85	1·82	0·0040*	5·75	3·72	0·007*	1·99	1·53	0·37
Tenant Creek	1·14	0·56	0·79	0·98	0·66	0·97	1·55	1·06	0·52
Communities < 50 km from Alice Springs	0·48	0·37	0·34	0·51	0·48	0·47	0·43	0·67	0·59
Communities 51 – 100 km from Alice Springs	5·61	2·96	0·0010*	7·74	5·56	0·004*	6·05	5·19	0·036*
Communities 101 – 300 km from Alice Springs	1·55	0·57	0·23	1·75	0·83	0·24	1·38	0·78	0·57
Communities 301 – 500 km from Alice Springs	1·25	0·49	0·56	1·48	0·78	0·46	1·11	0·64	0·85
Communities > 500 km from Alice Springs	1·33	0·60	0·53	1·22	0·68	0·73	0·73	0·61	0·71
Outside of Catchment Area	1·03	0·79	0·96	3·24	2·62	0·15	0·05	0·10	0·15
**Diagnosis**									
Fracture Upper Limb Excluding Hands	Baseline			Baseline			Baseline		
Fracture Hands	0·68	0·41	0·53	0·85	0·64	0·83	0·52	0·53	0·52
Fracture Lower Limb Excluding Foot and Ankle	1·49	0·98	0·54	1·74	1·48	0·52	2·49	2·26	0·32
Fracture Foot and Ankle	0·96	0·62	0·95	0·83	0·77	0·84	1·16	1·12	0·88
Fracture Spine and Pelvis	0·70	1·07	0·82	8·64	15·05	0·22	0·55	0·90	0·72
Lacerations Upper Limb (No Tendon Involvement)	2·61	1·12	0.025*	2·59	1·48	0·096	2·30	1·58	0·23
Lacerations Lower Limb (No Tendon Involvement)	0·35	0·54	0.500	0·39	0·67	0·59	0·75	1·20	0·86
Tendon Injury Upper Limb	0·38	0·35	0.298	0·60	0·60	0·61	0·34	0·53	0·49
Tendon Injury Lower Limb	1·09	1·68	0.957	1·48	2·35	0·80	1·83	3·29	0·74
Wound/ Soft Tissue Infection	5·83	2·40	< 0.0001*	6·59	3·65	0·001*	3·96	2·58	0·035*
Surgical Site Infection	0·46	0·71	0.615	0·38	0·60	0·54	11·84	23·00	0·20
Septic Arthritis	3·00	1·95	0.092	6·72	5·32	0·016*	0·56	0·89	0·72
Prosthetic Joint Infection	2·49	3·97	0.567	2·15	3·59	0·65	65·23	139·66	0·051
Osteomyelitis	4·80	3·20	0.019*	5·89	5·25	0·047*	2·44	2.68	0·42
SCFE	4·71	7·54	0.332	6·07	10·68	0·31	9·60	17.28	0·21
Perthe’s	7·72	12·66	0.213	101·64	235·22	0·046*	8·72	14.92	0·21
Other Acutes	3·46	2·44	0.079	0·72	1·56	0·88	5·32	4.52	0·050
Elective Major	1·29	1·29	0.796	1·89	3·02	0·69	1·94	2.14	0·55
Elective Minor	0·14	0·21	0.189	0·16	0·26	0·254	0·43	0.67	0·59

^*^Statistically significant finding; *SE* Standard Error, *SCFE* Slipped Capital Femoral Epiphysis

### Association between length of stay and self-discharge

Throughout the entire study period, the mean length of stay in Indigenous Australian patients who had self-discharged was 2·92 ± 5·39 days, whereas those who did not self-discharge had a mean of 3·88 ± 6·31 days. Logistic regression analysis showed that for every increased day admitted in hospital, the odds of self-discharge decreases by 3% [Odds Ratio (OR) = 0·97], holding all else constant. This was not statistically significant (*p* = 0·113).

We further analysed this association before and after the implementation of routine ALO collaboration. Our analysis showed a 1% decrease in the odds of self-discharge for every additional day in hospital (OR = 0·99) in Group 1. This was not statistically significant (*p* = 0·610). However, in Group 2, there was a statistically significant decrease in the odds of self-discharge, by 17%, with each increasing day of hospital stay (OR = 0·827, *p* = 0·040), holding all else constant.

## Discussion

To our knowledge this is the only study to quantify the impact of partnership between clinicians and Aboriginal Health Workers on the rate of self-discharge amongst Indigenous Australians within an Orthopaedic unit. Our findings demonstrate that routine inclusion of ALO within the Orthopaedic MDT is associated with a lower risk of self-discharge among Indigenous Australian patients.

Routine ALO input was associated with a smaller proportion of patients who self-discharged prior to definitive surgical or medical treatment, and completion of intravenous antibiotic course following surgery – which are arguably the more critical aspects of a patient’s care. We postulate that delays in allied health assessment may be a cause for patients self-discharging prior to clearance, however, this would need to be investigated further. As most patients admitted within our service would have experienced a similar treatment path (definitive surgical or medical treatment, completion of postoperative antibiotic treatment, allied health clearance, discharge medications), we did not modify our analysis. Our findings mirror that of O’Connor et. al, which showed a trend towards reduced self-discharge rates as interpreter use within the centre increased [20], as well as that of Taylor et. al., which showed reduced number of Indigenous self-discharges following creation of an Aboriginal Health Worker role [21]. Neither of these studies defined the precise role and extent of involvement of Aboriginal Health Workers.

Despite improvement in self-discharge rates following routine inclusion of ALO, they remain significantly higher than in non-Indigenous patients. On comparing risk factors for self-discharge between Group 1 and 2, patients living in Alice Springs town-camps, and those diagnosed with septic arthritis and osteomyelitis were no longer risk factors following routine inclusion of ALO. This change is encouraging, as it suggests that patients with these potentially more serious musculoskeletal conditions, were more likely to have comply with their recommended treatment in hospital. Although our statistical analysis may have suggested Perthe’s disease being a risk factor for self-discharge in Group 1, this is unlikely to be of clinical significance, as there was only a single patient diagnosed with this condition within the group. The association between length of stay and odds of self-discharge was analysed separately. Although our initial analysis of the data showed no association between length of stay and the odds of self-discharge, deeper analysis indicates a significantly lower odds of self-discharge in Group 2. This implies that patients requiring prolonged hospital treatment episodes experience a stronger beneficial relationship with ALO, generating a greater degree of trust in the system and improved patient compliance.

Despite introduction of ALO, patients residing in communities between 51 to 100 kms of Alice Springs continued to be a risk factor for self-discharge. It is possible that patients residing within this zone may feel isolated from their community, resulting in a leave event. Interestingly, patients residing in communities greater than 101 km from Alice Springs was not a risk factor for self-discharge. We speculate that patients living in these areas are still likely to experience isolation from their community but are unable to self-discharge due to a lack of access to transport back home. It is important to emphasise that these hypotheses would need to be assessed in a future qualitative analysis within our patient cohort. In addition to their residence category, patients of younger age, pensioners and those diagnosed with wound or soft tissue infections persisted as risk factors for self-discharge following the change in practice. Targeted health promotion campaigns, as well as special emphasis by clinicians on such patient demographics, may help reduce the risk of self-discharge.

Qualitative analysis on the subject of self-discharge suggests the decision to self-discharge to be a rational act of reclaiming personal autonomy and having agency over ones healthcare decisions, likely stemming from unmet needs [22]. In most patients, inability to delegate responsibilities inherent to social or cultural roles and financial constraints in combination with perceived improvement in ones’ health, often precipitate a leave event [8, 23, 24]. In our experience, ALO are often related to patients through bonds of kinship. This enables trust and confidence in the interactions between patient and the treating MDT, empowering patients to voice their concerns surrounding care, as well as openly disclose often competing priorities, for example childcare, which may otherwise be undeclared. This allows for the development of a more comprehensive management strategy and allows for early involvement of other services, such as social work, to potentially mitigate competing priorities.

Unlike most non-Indigenous patients, Indigenous patients have everyday lived experiences of personal and institutional bias and racism [22], and unsurprisingly many may anticipate and perceive discrimination regardless of its presence [25]. Healthcare facilities in Australia often embody policies shaped by Western perception of health, which are often focussed on the recognition and treatment of disease [26]. In contrast, Indigenous people have a more holistic view of health, which encompasses the physical wellbeing of the individual in combination with the social, emotional, and cultural well-being of one’s whole community [27]. In this regard, ALOs help provide cultural brokerage, allow for shared decision-making process, and provide a culturally safe environment for patients, encouraging patients’ requests for services outside the scope of Western medicine. This may include the service of Ngangkaris (Traditional Healers), who perform healing rituals. Recent evidence have proposed self-discharge as a marker of surgical cultural competency and cultural safety [28]. In the context of our study, routine ALO collaboration is a positive step towards cultural competency. However, we acknowledge that there are further measures that could be taken, such as increasing employment of Indigenous health care providers and cultural safety training for staff members, which may continue to reduce the rates of self-discharge.

The use of ALO also helps improve communication between the patient and their healthcare providers. It has been well documented in literature that Indigenous patients often feel frustrated and misunderstood, and even intimidated by healthcare professionals [15, 29]. In an interview with an Indigenous patient for a qualitative study by Kerrigan et. al, it was remarked that he could comprehend about half of what was explained to him [15]. Although ALO are different from trained interpreters, most of the ALO in our hospital are proficient multilinguists. On rare occasions, where the ALO felt that they could not communicate in the patient’s language, this was highlighted, and alternative arrangements were made. It is postulated that a patients’ comprehensive understanding of their disease, treatment, and progress, would better incline patients to engage fully in their treatment and reduce risk of their self-discharge. Previous studies have shown improved patient engagement in rehabilitation following close partnership between specialist nursing staff and ALO [30].

Our study has a few limitations. Firstly, by virtue of its retrospective design the accuracy of our data was dependent upon the quality of data collection and entry during each treatment episode. Secondly, although we used self-discharge as a surrogate tool for improvement in service provision, there is potential that some patients may have self-discharged for reasons other than their dissatisfaction with our service. Analysis of non-Indigenous patients suggest that there were no significant changes in rates of self-discharge between the two groups. This implies that the change in self-discharge is likely due to implementation of routine ALO. Thirdly, there may be other changes within the hospital structure, apart from our ALO inclusion across the study period, which may have influenced self-discharge rates. Fourthly, we were unable to quantify the harm or increase in healthcare burden inflicted by self-discharge. It is plausible to assume that at least some self-discharged patients may have developed early complications or disease recurrence and presented to local health clinics, whilst others could present with late complications, either to our service or elsewhere.

Another limitation of our study was that there was also no definition of an Indigenous patient. Given the nature of the study design, patients were classified as Indigenous or non-Indigenous based upon self-identification. We were not able to further classify our patients into Aboriginal, Torres Strait Islander, or Aboriginal and Torres Strait Islander people. Furthermore, we did not have data on the role of caregivers or families in informing our patient’s self-discharge. In addition to this, some patients in Group 2 would not have exposure to an ALO, as this service would have only been provided on weekdays (excluding public holidays). Of the 42 patients who self-discharged in Group 2, ten patients have done so over the weekend, and hence had no exposure to ALO. Lastly, the regression model of risk factors only considered independent variables that would be available from routinely collected patient data and may not represent the full spectrum of probable causes.

In conclusion, routine incorporation of ALO within the Orthopaedic MDT was associated with a significantly lower risk of self-discharge among Indigenous Australian patients. This entails no significant added costs, simply a shift in mind-set within the Orthopaedic MDT to fully engage and empower the existing workforce of ALO within the hospital. Patients were less likely to leave prior to completion of the most critical aspects of their care. These results are likely generalisable to other centres within Australia. The potential for future out of business hours, weekend and public holiday ALO service provision in ASH is perceived by the authors as a likely positive driver for further improvement in patient care. Targeted health promotion campaigns may aid in further lowering rates of self-discharge. Further research into the risk factors for self-discharge, as well as qualitative research on patient perception of our proposed healthcare model should be considered.

## Data Availability

Available on request.

## Abbreviations

ALO: Aboriginal Liaison Officers
ASH: Alice Springs Hospital
MDT: Multi-Disciplinary Team
OR: Odds Ratio
RR: Risk Ratio
STROBE: Strengthening the Reporting of Observational Studies in Epidemiology
